# Prognostic significance of bladder neck involvement in non‐muscle‐invasive bladder cancer: A SEER database analysis with 19,919 patients

**DOI:** 10.1002/cam4.4219

**Published:** 2021-08-23

**Authors:** Xiangpeng Zhan, Ju Guo, Luyao Chen, Wen Deng, Xiaoqiang Liu, Ke Zhu, Weipeng Liu, Bin Fu

**Affiliations:** ^1^ Department of Urology The First Affiliated Hospital of Nanchang University Nanchang Jiangxi Province China; ^2^ Jiangxi Inst Urol Nanchang Jiangxi P. R. China

**Keywords:** bladder neck involvement, prognosis, SEER database, urinary bladder

## Abstract

**Purpose:**

To study prognostic values of bladder neck involvement (BNI) and survival outcomes in non‐muscle‐invasive bladder cancer (NMIBC).

**Method and materials:**

The national Surveillance, Epidemiology, and End Results database (2004–2015) was applied to gain further insight into the prognostic values of BNI and 19,919 patients diagnosed with NMIBC were included in our study. We used the Kaplan–Meier method with the log‐rank test and subgroup analyses to evaluate cancer‐specific survival (CSS) and overall survival (OS). In addition, the multivariable Cox proportional hazard model and propensity score matching (PSM) were utilized.

**Results:**

In all, 3446 patients with BNI and 16,473 patients with sites except for bladder neck were enrolled in our study. Compared with other sites, a tendency toward a higher proportion of higher grade (*p* < 0.001), bigger tumor size (*p* < 0.001), and more patients with T1 and Tis stage (*p* < 0.001) was seen in BNI group. After 1:1 PSM, 3425 matched pairs were selected. Under the survival analyses, the BNI group had a lower survival probability in both OS (*p* = 0.0056) and CSS analyses (*p* < 0.0001) in NMIBC patients. However, in the subgroup analysis, only observed in the Ta and T1 stage in terms of CSS (all *p* < 0.05), and patients with Tis stage failed to show statistical survival differences (*p* > 0.05). In addition, subgroups stratified by tumor size and grade all revealed poor prognosis of BNI in NMIBC patients. Moreover, better survival outcomes of OS were observed in BNI patients who received radical cystectomy (*p* = 0.02) or chemotherapy (*p* < 0.001) multivariable Cox regression after PSM revealed that the BNI group had a higher risk of overall mortality (OM) (BNI vs. other sites hazards ratios [HR]: 1.127, 95% CI: 1.154–1.437, *p* < 0.001) and cancer‐specific mortality (CSM) (BNI vs. other sites HR: 1.127, 95% CI: 1.039–1.223, *p* < 0.001), while before PSM, similar situations were only existed in CSM (BNI vs. other sites HR: 1.288, 95% CI: 1.154–1.437, *p* < 0.001).

**Conclusions:**

The prognosis of BNI was poorer than that of the other sites. BNI was an independent risk factor for OM and CSM in patients with NMIBC, especially for those with Ta or T1 stage.

## Graphical abstract

Bladder neck involvement (BNI) prognosis is poorer than that of the other sites. BNI is an independent prognostic factor for ACM and cancer‐specific mortality in patients with non‐muscle‐invasive bladder cancer.

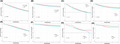

## 1 INTRODUCTION

Bladder cancer is the second commonly diagnosed malignancy and the most prevalent cancers worldwide, with 549,393 new cases reported in 2018.^1^ In these patients, approximately 75% of newly confirmed urinary bladder neoplasms cases consist of non‐muscle‐invasive bladder cancer (NMIBC),^2^ which included Ta, T1, and Tis stage, and had a high rate of recurrence but low mortality. The European Organization for Research, Treatment of Cancer (EORTC) and Club Urologico Espanol de Tratamiento Oncologico (CUETO) risk scores had been widely used to predict oncological outcomes of NMIBC patients and a cohort analysis including 322 NMIBC patients obtained the result that EORTC showed the best recurrence and progression prediction.^3^ They used a scoring system based on several clinical and pathological characteristics, including the number of tumors, tumor diameter, prior recurrence rate, T category, concurrent CIS category, and grade. However, the predictive accuracy of these models remained unsatisfactory, and it might be due to inappropriate predictors. Thus, the need for better disease outcome prediction demanded to include more relevant factors to make these models more precise.

Over the course of the past 30 years, several retrospective studies reported that bladder neck involvement (BNI) was an independent risk factor of recurrence and progression in NMIBC patients.^4^, ^5^, ^6^, ^7^ A new predictive scoring model for progression combining with BNI^8^ represented a higher c‐index of 0.59 than the EORTC (0.57) and CUETO (0.50) models. William T. Stephenson, MD^9^ first raised significant differences in potential neoplastic growth among the different subsites within the urinary bladder. A significantly poorer prognosis of tumor on bladder neck by survival analysis was proposed in this study. Obviously, these studies intended to prove that BNI might have a poorer prognosis of oncological outcome than the other sites of bladder. However, some controversial results were presented in some research. For instance, Hamed Ahmadi^10^ got the result that posterior wall of bladder tumors had worse overall survival (OS) than other sites. A retrospective study based on National Cancer Database (2004–2015) revealed that patients with trigone of bladder involvement might have poorer OS for patients following chemoradiotherapy.^11^ Unfortunately, they could not produce consistent results, and all were limited by their small sample sizes, different study populations, heterogeneous patient cohorts, and various definitions of tumor location. The influence of BNI on disease outcome, especially for survival outcome, was rarely studied in patients with NMIBC. To better understand the prognostic significance of BNI especially for survival outcome in NMIBC patients, we used the Surveillance, Epidemiology, and End Results (SEER) database (2004–2015) to provide a new recognition of BNI and survival outcomes between BNI and other sites in NMIBC patients.

## 2 MATERIALS AND METHODS

### 2.1 Patient database and study population

Patient data were obtained from the SEER which collected patient demographic and cancer data of the US population. All patients were included if they met the following criteria (*n* = 28,750): (a) Year of diagnosis: 2004–2015; (b) Pathological diagnosis (not include Clinical diagnosis only, radiography without microscopic confirm direct visualization without microscopic confirmation, method not specified); (c) T stage include: Ta, Tis, and T1 stage. (d) N; M stage: N0; M0; (e) Histology behavior: transitional cell carcinoma. The following exclusion terms were showed: (a) Unclear tumor location or too few cases (*n* = 8802): overlapping subsites, bladder NOS and Urach (b) unknown survival status (*n* = 29). A total of 19,919 patients were included in our retrospective study.

### 2.2 Definition of variables

Demographic characteristics contained age at diagnosis, gender, race, and marital status. Disease characteristics included tumor location, T status, grade, tumor size, and number of tumors. Treatment information included the surgery approach, radiation recode, and chemotherapy recode. The race was classified into four categories: White, Black, Asian/Pacific Islander, and American Indian/Alaskan Native. Marital status was defined as married, single, widowed or divorced, and unknown. Tumor location was coded based on ICD‐O‐3 topography as follows: C67.0 trigone, C67.1, dome; C67.2, lateral wall; C67.3, anterior wall; C67.4, posterior wall; C67.5, bladder neck; and C67.6, ureteral orifice. Other sites group comprised trigone, dome, lateral wall, anterior wall, posterior wall, and ureteral orifice. The American Joint Committee on Cancer 6th edition was used to identify pathological T status. T stages were divided into subgroups as Ta, Tis, and T1. Tumor grade was divided into two groups: grade I/grade II and grade III/grade IV. Tumor size was recorded as the largest dimension of the primary tumor, divided into two subgroups, included ≤3 and >3 cm. The total number of tumors was the maximum sequence number in the bladder through SEER. According to the “RX Summ‐Surg Prim Site (1998+)” column in the SEER database, the surgical method was divided into TURBT, partial cystectomy, radical cystectomy, and pelvic exenteration. Other variables included: (1) Radiation recode (no/unknown, yes); (2) Chemotherapy recode (no/unknown, yes).

### 2.3 Endpoints

The primary endpoints were overall mortality (OM) and cancer‐specific mortality (CSM). OM referred to the death of patients, including any cause. The CSM rate referred to only those patients who died of bladder cancer were included when the cause of death was confirmed. Survival time was defined as the duration from the initial diagnosis to death from any cause or to the last follow‐up. Any patient could be recoded to alive if they died after the follow‐up cut‐off date.

### 2.4 Statistical analysis

All statistical analyses and propensity score matching (PSM) were carried out by SPSS version 22.0. Patients’ baseline characteristics were evaluated to confirmed whether there were significant differences in the distribution of patients. Two‐sample *t*‐tests and chi‐square test were used for categorical variables and continuous variables to verify heterogeneity between two groups. All results’ *p*‐values were two‐tailed, and a *p* < 0.050 was recognized as significant. R and RStudio were applied to construct OS and cancer‐specific survival (CSS) curves using the Kaplan–Meier method and the log‐rank test. Subgroup analysis was performed for OS and CSS of NMIBC patients stratified by T stage (Ta, T1, and Tis), tumor size (<3 and >3 cm), and tumor grade (Grade Ⅰ/Ⅱ and Grade Ⅲ/Ⅳ). In addition, subgroups stratified by surgery (TURBT and RC), radiotherapy (No/unknown and Yes) and chemotherapy (No/unknown and Yes) were divided to obtain more information on treatment for BNI patients. Univariate and multivariate Cox regression analysis were used to analyze the impact of tumor location on prognosis, and the results were presented as hazards ratios (HR) and 95% confidence intervals (95% CI). In univariate analysis, variables with a *p*‐value of 0.05 or less were included as candidate variables in the multivariate analysis. The multivariate Cox regression model adjusted age, race, marital status, grade, tumor location, T stage, tumor size, number of tumors, radiation recode, and chemotherapy recode for OM and age, race, grade, tumor location, T stage, tumor size, number of tumors, surgery, radiation recode, and chemotherapy recode for CSM. PSM was carried out with SPSS to adjust the potential baseline further confounding factors model. For PSM, patients receiving bladder neck or other sites were matched 1:1 with a caliper set at 0.001. Greedy method and a logistic model with nearest neighbor algorithm were used.

## 3 RESULTS

### 3.1 Baseline characteristics of the research population before and after PSM

**FIGURE 1:**
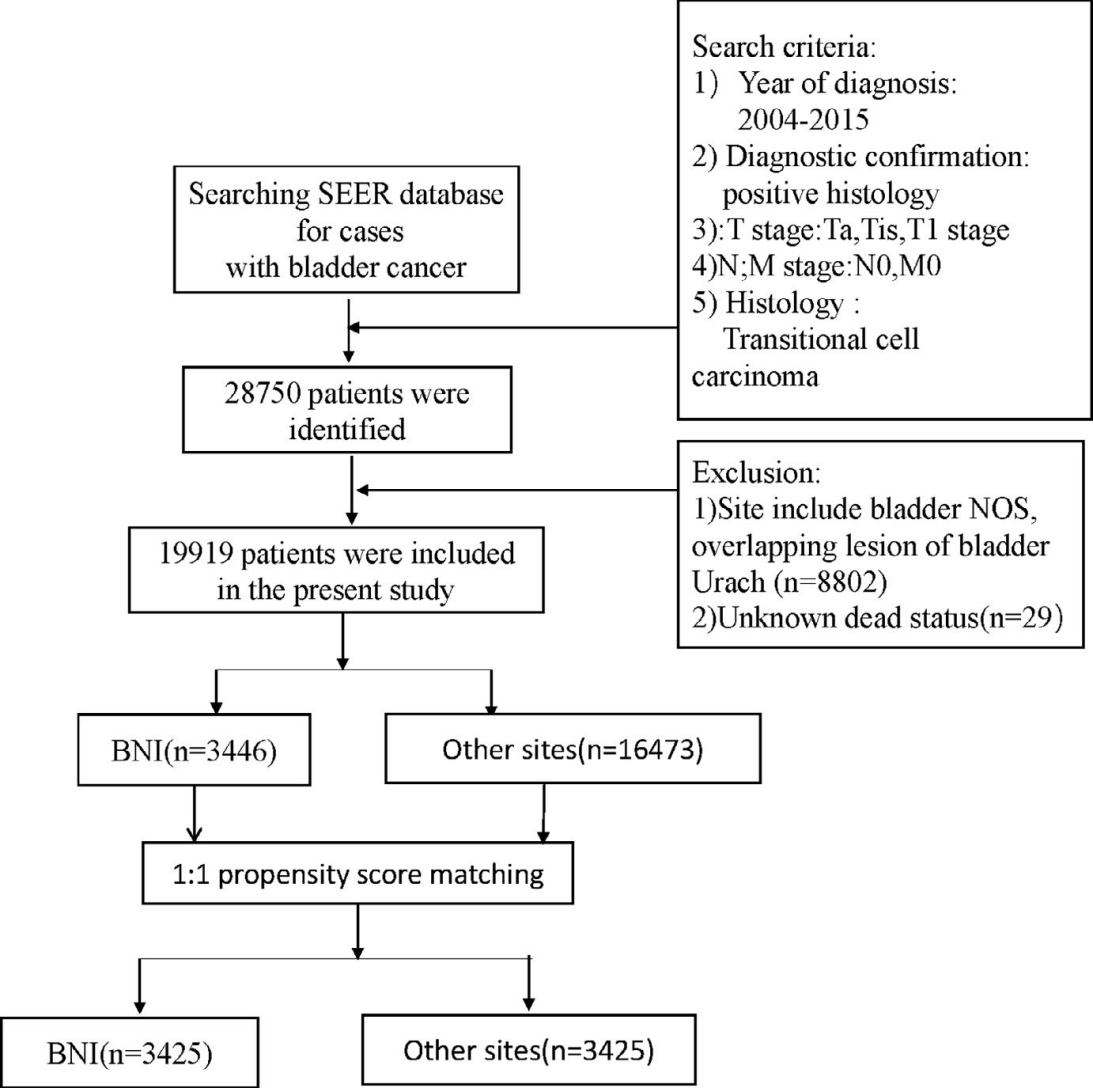
Flow‐chart showing the procedure used to identify patients with NMIBC in the SEER database from 2004 to 2015. NMIBC, non‐muscle‐invasive bladder cancer; SEER, Surveillance, Epidemiology, and End Results

A total of 19,919 NMIBC patients from the SEER database (2004–2015) were finally included in our study (Figure 1). There were 16,473 patients in the other sites group and 3446 patients in the BNI group. Before PSM, no significant differences in terms of race (*p* = 0.389), marital status (*p* = 0.968), chemotherapy recode (*p* = 0.364), and number of tumor (*p* = 0.183) were observed between two different two groups (all *p* > 0.050). However, patients with bladder neck tumors tended to be male (78.4% vs. 76.6%, *p* = 0.021) and old (71.01 ± 10.87 vs. 70.04 ± 11.33 *p* < 0.001). Patients in the BNI group had higher rate of grade Ⅲ and grade IV (55.9% vs. 45.0%, *p* < 0.001), T1 stage (42.9% vs. 33.8%, *p* < 0.001) and Tis stage (3.3% vs. 45.0%, *p* < 0.001). The surgery approach was significantly different between two groups (*p* < 0.001), and patients in the BNI group seemed to undergo radical cystectomy (1.5% vs. 0.6%) and pelvic exenteration (1.6% vs. 0.7%). Moreover, radiotherapy was more common receiving in BNI patients (1.0% vs. 0.5%, *p* < 0.001). As for tumor size, BNI group had higher proportion of big size in >3 cm subgroup (51.6% vs. 30.5% *p* < 0.001). After 1:1 PSM adjusting for sex, race, grade, TNM stages, surgery, and tumor size. However, there were still differences in race, grade, and surgery between the two groups (Table 1).

**TABLE 1:** Clinicopathological features between BNI and other sites before and after propensity score matching

Variables	PSM			No PSM		
	BNI (*n* = 3425)	Other sites (*n* = 3425)	*p*‐value	BNI (*n* = 3446)	Other sites (*n* = 16,473)	*p*‐value
Age (year) mean	70.027 ± 10.88	71.101 ± 10.97	0.787	71.01 ± 10.87	70.04 ± 11.33	<0.001*
Sex			0.131			0.021*
Male	2686 (78.4%)	2634 (76.9%)		2703 (78.4%)	12,620 (76.6%)	
Female	739 (21.6%)	791 (23.1%)		743 (21.6%)	3853 (23.4%)	
Race			0.004*			0.389
White	3093 (90.3%)	3070 (89.6%)		3112 (90.3%)	14,910 (90.5%)	
Black	184 (5.4%)	158 (4.6%)		184 (5.3%)	794 (4.8%)	
AIAN	14 (0.4%)	8 (0.2%)		14 (0.4%)	55 (0.3%)	
API	134 (3.9%)	189 (5.5%)		136 (3.9%)	714 (4.3%)	
Marital status			0.986			0.608
Married	11,758 (58.9%)	239 (57.9%)		2118 (61.5%)	10,211 (62.0)	
Single	2136 (10.7%)	56 (13.6%)		340 (9.9%)	1685 (10.2%)	
Widowed/divorced	4576 (22.9%)	95 (23.0%)		798 (23.2%)	3742 (22.7%)	
Unknown	1487 (7.5%)	23 (5.6%)		190 (5.5%)	835 (5.1%)	
Grade			0.057			<0.001*
Grade I or Grade II	1520 (44.4%)	1442 (42.1%)		1521 (44.1%)	9056 (55.0%)	
Grade III or Grade IV	1905 (55.6%)	1983 (57.9%)		1925 (55.9%)	7417 (45.0%)	
T stage			0.031*			<0.001*
Ta	1849 (54.0%)	1956 (57.1%)		1852 (53.7%)	10,627 (64.5%)	
Tis	113 (3.3%)	100 (2.9%)		114 (3.3%)	421 (2.6%)	
T1	1463 (42.7%)	1369 (40.0%)		1480 (42.9%)	5425 (33.9%)	
Surgery			0.094			<0.001*
TURBT	3318 (96.9%)	3310 (96.6%)		3322 (96.4%)	16,162 (98.1%)	
Partial cystectomy	17 (0.5%)	28 (0.8%)		17 (0.5%)	87 (0.5%)	
Radical cystectomy	47 (1.4%)	33 (1.0%)		52 (1.5%)	101 (0.6%)	
Pelvic exenteration	43 (1.3%)	54 (1.6%)		55 (1.6%)	123 (0.7%)	
Radiation recode			0.551			<0.001*
No/unknown	3392 (99.0%)	3387 (98.9%)		3410 (99.0%)	16,394 (99.5%)	
Yes	33 (1.0%)	38 (1.1%)		36 (1.0%)	79 (0.5%)	
Chemotherapy recode			<0.001*			0.364
No/unknown	2534 (74.0%)	2373 (69.3%)		2545 (73.9%)	12,288 (74.6%)	
Yes	891 (26.0%)	1052 (30.7%)		901 (26.1%)	4185 (25.4%)	
Tumor size			0.828			<0.001*
≤3 cm	1667 (48.7%)	1676 (48.9%)		1667 (48.4%)	11,446 (69.5%)	
>3 cm	1758 (51.3%)	1749 (51.1%)		1779 (51.6%)	5027 (30.5%)	
Number of tumors			0.054			0.183
1	2148 (62.7%)	2056 (60.0%)		2158 (62.6%)	10,633 (64.5%)	
2	953 (27.8%)	985 (28.8%)		958 (27.8%)	4368 (26.5%)	
3	252 (7.4%)	300 (8.8%)		258 (7.5%)	1136 (6.9%)	
>4	72 (2.1%)	84 (2.5%)		72 (2.1%)	336 (2.0%)	

Abbreviations: AIAN, American/Indian/Alaska/Native; API, Asian/Pacific Islander; BNI, bladder neck involvement; PSM, propensity score matching; TURBT, transurethral bladder tumor resection.

* Statistically significant.

### 3.2 Multivariable analysis of risk factors for CSM and OM before and after PSM

In survival analyses, the median follow‐up time was 46.94 months. Univariate analyses (Tables 2 and 3) showed that age, race, marital status, grade, primary tumor site, T stages, tumor size, number of tumors, radiotherapy, and chemotherapy were all associated with poorer OM before and after PSM (all *p* < 0.05). As for CSM (Tables 3 and 5), race, marital status, grade, T stage, surgery, radiotherapy, chemotherapy, tumor size, and number of tumors were correlated with it. After adjusting for age, race, marital status, grade, primary tumor site, T stage, tumor size, number of tumors surgery, radiotherapy, and chemotherapy for OM and CSM. Before PSM, the multivariable Cox proportional hazard models (Tables 4 and 5) revealed that BNI was an independent risk factor for CSM (BNI vs. other sites HR: 1.288, 95% CI: 1.154–1.437, *p* < 0.001) while no difference in OM was witnessed between two groups (HR = 1.043, 95% CI = 1.039–1.223, *p* = 0.004). However, statistical differences (Tables 2 and 3) on OM (BNI vs. other sites HR: 1.127, 95% CI: 1.154–1.437, *p* < 0.001) and CSM (BNI vs. other sites HR: 1.127, 95% CI: 1.039–1.223, *p* < 0.001) were both observed. In addition (Tables 2 and 3), age, race, and marital status as demographic characteristics were independent prognostic factors for both OM and CSM for NMIBC patients in the multivariate Cox regression analysis (all *p* < 0.05). Similar situations were found in grade, T stage, tumor size, and number of tumor. Interestingly, patients who received radiotherapy had worse prognosis of ACM and CSM than those not (HR = 2.038, 95% CI = 1.555–2.671, *p* < 0.001 for OM; HR = 1.769, 95% CI = 1.110–2.692, *p* = 0.001 for CSM, respectively) while receiving chemotherapy was a protective factor of OM (HR = 0.785, 95% CI = 0.677–0.878, *p* < 0.001) in NMIBC patients.

**TABLE 2:** Univariate and multivariate regression analyses for OM after PSM

Characteristic	Univariate			Multivariate		
	HR	95% CI	*p*	HR	95% CI	*p*
Age (year)	1.082	1.076–1.087	<0.001*	1.073	1.068–1.079	<0.001*
Sex						
Female	Ref.					
Male	1.046	0.947–1.154	0.377			
Race						
White	Ref.			Ref.		
Black	1.162	0.973–1.387	0.097	1.215	1.046–1.496	0.014
AIAN	1.028	0.489–2.158	0.943	1.137	0.541–2.393	0.735
API	0.786	0.637–0.971	0.025*	0.926	0.749–1.144	0.474
Marital status						
Married	Ref.					
Single	1.074	0.928–1.243	0.338	1.369	1.182–1.585	<0.001*
Widowed/divorced	1.727	1.576–1.892	<0.001*	1.321	1.204–1.450	<0.001*
Grade						
Grade I or Grade II	Ref.			Ref.		
Grade III or Grade II	1.646	1.511–1.792	<0.001*	1.154	1.046–1.274	0.004*
Primary tumor site						
Other sites				Ref.		
Bladder neck	1.121	1.034–1.216	0.006*	1.127	1.039–1.223	0.004*
T stage						
Ta	Ref.			Ref.		
Tis	1.255	0.985–1.598	0.066	1.052	0.825–1.341	0.685
T1	1.797	1.654–1.951	<0.001*	1.449	1.316–1.596	<0.001*
Surgery						
TURBT	Ref.					
Partial cystectomy	0.877	0.518–1.483	0.624			
Radical cystectomy	1.049	0.728–1.514	0.796			
Pelvic exenteration	1.077	0.764–1.519	0.671			
Radiation recode						
No/unknown	Ref.			Ref.		
Yes	3.711	2.892–4.917	<0.001*	2.038	1.555–2.671	<0.001*
Chemotherapy recode						
No/unknown	Ref.					
Yes	0.734	0.667–0.808	<0.001*	0.785	0.677–0.878	<0.001*
Tumor size						
≤3 cm	Ref.			Ref.		
>3 cm	1.374	1.266–1.491	<0.001*	1.277	1.168–1.617	<0.001*
Number of tumors						
1	Ref.			Ref.		
2	1.731	1.582–1.893	<0.001*	1.453	1.327–1.591	<0.001*
3	2.337	2.058–2.655	<0.001*	1.789	1.573–2.034	<0.001*
>4	2.526	2.042–3.124	<0.001*	1.922	1.552–2.381	<0.001*

Adjust for: age, race, marital status, Grade, tumor location, T stage, tumor size, number of tumor, chemotherapy recode, radiation recode.

Abbreviations: AIAN, American/Indian/Alaska/Native; API, Asian/Pacific Islander; CI, confidence interval; HR, hazard ratio; OM, overall mortality; PSM, propensity score matching; Ref., reference; TURBT, transurethral bladder tumor resection.

* Statistically significant.

**TABLE 3:** Univariate and multivariate regression analyses for CSM after PSM

Characteristic	Univariate			Multivariate		
	HR	95% CI	*p*	HR	95% CI	*p*
Age (year)	1.069	1.060–1.078	<0.001*	1.007	0.998–1.015	0.110
Sex						
Female	Ref.					
Male	0.987	0.833–1.169	0.879			
Race						
White	Ref.			Ref.		
Black	1.577	1.203–2.066	0.001*	1.379	1.047–1.817	0.022*
AIAN	1.320	0.425–4.130	0.631	1.895	0.605–5.930	0.272
API	0.877	0.615–1.251	0.469	1.076	0.751–1.541	0.690
Marital status						
Married	Ref.					
Single	1.380	1.095–1.740	0.006	1.416	1.119–1.793	0.004*
Widowed/divorced	1.700	1.447–1.997	<0.001*	1.092	0.927–1.287	0.292
Grade						
Grade I or Grade II	Ref.			Ref.		
Grade III or Grade II	3.239	2.722–3.853	<0.001*	1.733	1.419–2.116	<0.001*
Primary tumor site						
Other sites				Ref.		
Bladder neck	1.409	1.222–1.625	<0.001*	1.383	1.198–1.598	<0.001*
T stage						
Ta	Ref.			Ref.		
Tis	1.664	1.065–2.600	0.025*	1.377	0.877–2.162	0.164
T1	3.311	2.839–3.863	<0.001*	1.703	1.423–2.037	<0.001*
Surgery						
TURBT	Ref.			Ref.		
Partial cystectomy	1.984	1.063–3.704	0.031*	1.817	0.968–3.409	0.063
Radical cystectomy	1.833	1.117–3.008	0.017*	1.118	0.673–1.857	0.666
Pelvic exenteration	2.092	1.355–3.228	0.001*	1.729	1.110–2.692	0.015
Radiation recode						
No/unknown	Ref.			Ref.		
Yes	6.964	4.958–9.782	<0.001*	1.769	1.110–2.692	0.001*
Chemotherapy recode						
No/unknown	Ref.					
Yes	0.786	0.667–0.927	0.004*	0.994	0.841–1.174	0.943
Tumor size						
≤3 cm	Ref.			Ref.		
>3 cm	1.859	1.604–2.154	<0.001*	1.410	1.212–1.640	<0.001*
Number of tumors						
1	Ref.			Ref.		
2	1.561	1.332–1.829	<0.001*	0.931	0.793–1.094	0.387
3	2.298	1.847–2.860	<0.001*	0.988	0.792–1.234	0.918
>4	2.917	2.072–4.160	<0.001*	1.118	0.841–1.679	0.328

Adjust for: age, race, marital status, grade, tumor location, t stage, tumor size, number of tumor, surgery, chemotherapy recode, radiation recode.

Abbreviations: AIAN, American/Indian/Alaska/Native; API, Asian/Pacific Islander; CI, confidence interval; CSM, cancer‐specific mortality; HR, hazard ratio; PSM, propensity score matching; Ref., reference; TURBT, transurethral bladder tumor resection.

* Statistically significant.

**TABLE 4:** Univariate and multivariate regression analyses for OM before PSM

Characteristic	Univariate			Multivariate		
	HR	95% CI	*p*	HR	95% CI	*p*
Age (year)	1.084	1.081–1.087	<0.001*	1.076	1.072–1.079	<0.001*
Sex						
Female	Ref.					
Male	1.057	0.995–1.123	0.070			
Race						
White	Ref.			Ref.		
Black	1.072	0.957–1.200	0.229	1.185	1.058–1.328	0.003*
AIAN	0.693	0.410–1.171	0.171	0.799	0.473–1.351	0.403
API	0.721	0.625–0.830	<0.001*	0.781	0.677–0.900	0.001
Marital status						
Married	Ref.					
Single	1.380	1.095–1.740	0.006	1.324	1.209–1.450	<0.001*
Widowed/divorced	1.700	1.447–1.997	<0.001*	1.314	1.241–1.391	<0.001*
Grade						
Grade I or Grade II	Ref.			Ref.		
Grade III or Grade II	1.632	1.552–1.717	<0.001*	1.160	1.095–1.230	<0.001*
Primary tumor site						
Other sites				Ref.		
Bladder neck	1.212	1.138–1.290	<0.001*	1.043	0.978–1.112	0.196
T stage						
Ta	Ref.			Ref.		
Tis	1.223	1.047–1.430	0.011*	1.060	0.906–1.239	0.467
T1	1.721	1.636–1.811	<0.001*	1.374	1.295–1.457	<0.001*
Surgery						
TURBT	Ref.			Ref.		
Partial cystectomy	0.988	0.705–1.384	0.944	1.817	0.968–3.409	0.063
Radical cystectomy	1.097	0.841–1.430	0.495	1.118	0.673–1.857	0.666
Pelvic exenteration	1.142	0.884–1.476	0.310	1.729	1.110–2.692	0.015
Radiation recode						
No/unknown	Ref.			Ref.		
Yes	4.118	3.336–5.084	<0.001*	2.239	1.809–2.771	<0.001*
Chemotherapy recode						
No/unknown	Ref.					
Yes	0.753	0.708–0.801	<0.001*	0.783	0.735–0.833	<0.001*
Tumor size						
≤3 cm	Ref.			Ref.		
>3 cm	1.362	1.294–1.433	<0.001*	1.233	1.170–1.300	<0.001*
Number of tumors						
1	Ref.			Ref.		
2	1.847	1.748–1.952	<0.001*	1.489	1.417–1.583	<0.001*
3	2.577	2.377–2.794	<0.001*	1.926	1.775–2.088	<0.001*
>4	2.633	2.229–3.014	<0.001*	1.929	1.684–2.210	<0.001*

Adjust for: age, race, marital status, grade, histology behavior, tumor location, T stage, tumor size, number of tumor, chemotherapy recode, radiation recode.

Abbreviations: AIAN, American/Indian/Alaska/Native; API, Asian/Pacific Islander; CI, confidence interval; HR, hazard ratio; OM, overall mortality; PSM, propensity score matching; Ref., reference; TURBT, transurethral bladder tumor resection.

* Statistically significant.

**TABLE 5:** Univariate and multivariate regression analyses for CSM before PSM

Characteristic	Univariate			Multivariate		
	HR	95% CI	*p*	HR	95% CI	*p*
Age (year)	1.077	1.071–1.083	<0.001*	1.065	1.059–1.071	<0.001*
Sex						
Female	Ref.					
Male	0.912	0.818–1.016	0.070			
Race						
White	Ref.			Ref.		
Black	1.332	1.098–1.616	0.229	1.356	1.116–1.648	0.002*
AIAN	1.051	0.472–2.334	0.171	1.235	0.553–2.759	0.607
API	0.896	0.703–1.142	<0.001*	0.977	0.766–1.246	0.850
Marital status						
Married	Ref.					
Single	1.194	1.019–1.400	0.006	1.557	1.326–1.827	<0.001*
Widowed/divorced	1.772	1.595–1.969	<0.001*	1.371	1.232–1.526	<0.001*
Grade						
Grade I or Grade II	Ref.			Ref.		
Grade III or Grade II	3.520	3.164–3.917	<0.001*	2.757	2.473–3.074	<0.001*
Primary tumor site						
Other sites				Ref.		
Bladder neck	1.696	1.523–1.889	<0.001*	1.288	1.154–1.437	<0.001*
T stage						
Ta	Ref.			Ref.		
Tis	1.777	1.317–2.397	0.011*	1.380	1.022–1.863	0.036
T1	3.483	3.155–3.844	<0.001*	2.063	1.834–2.390	<0.001*
Surgery						
TURBT	Ref.					
Partial cystectomy	2.253	1.465–3.464	0.944			
Radical cystectomy	2.052	1.413–2.982	0.495			
Pelvic exenteration	2.270	1.608–3.203	0.310			
Radiation recode						
No/unknown	Ref.			Ref.		
Yes	8.833	6.750–11.559	<0.001*	3.430	2.606–4.514	<0.001*
Chemotherapy recode						
No/unknown	Ref.					
Yes	0.757	0.675–0.850	<0.001*	0.733	0.653–0.824	<0.001*
Tumor size						
≤3 cm	Ref.			Ref.		
>3 cm	1.976	1.799–2.170	<0.001*	1.490	1.352–1.642	<0.001*
Number of tumors						
1	Ref.			Ref.		
2	1.631	1.469–1.811	<0.001*	1.358	1.222–1.509	<0.001*
3	2.482	2.137–2.882	<0.001*	1.875	1.613–2.180	<0.001*
>4	2.424	1.874–3.136	<0.001*	1.834	1.416–2.376	<0.001*

Adjust for: age, race, marital status, grade, histology behavior, tumor location, t stage, tumor size, number of tumor, chemotherapy recode, radiation recode.

Abbreviations: AIAN, American/Indian/Alaska/Native; API, Asian/Pacific Islander; CI, confidence interval; CSM, cancer‐specific mortality; HR, hazard ratio; PSM, propensity score matching; Ref., reference; TURBT, transurethral bladder tumor resection.

* Statistically significant.

### 3.3 Survival analyses and subgroup analysis results of BNI on OS and OSS

**FIGURE 2:**
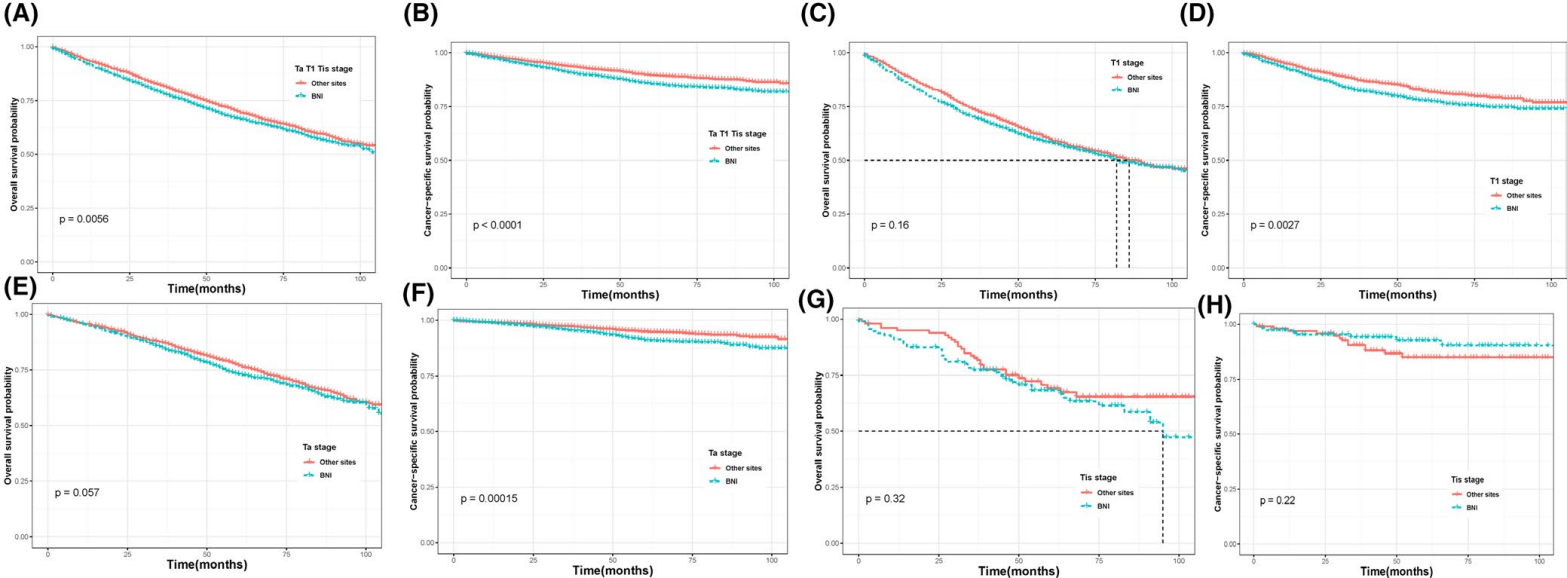
Effect of the bladder neck involvement on cancer‐specific survival (B, D, F, and H) and overall survival (A, C, E, and G) in non‐muscle‐invasive bladder cancer patients stratified by pathological T stage after propensity score matching

**FIGURE 3:**
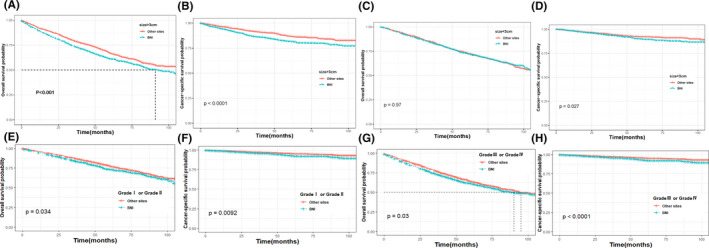
Effect of the bladder neck involvement on cancer‐specific survival (B, D, F, and H) and overall survival (A, C, E, and G) in non‐muscle‐invasive bladder cancer patients stratified by tumor size and grade after propensity score matching

**FIGURE 4:**
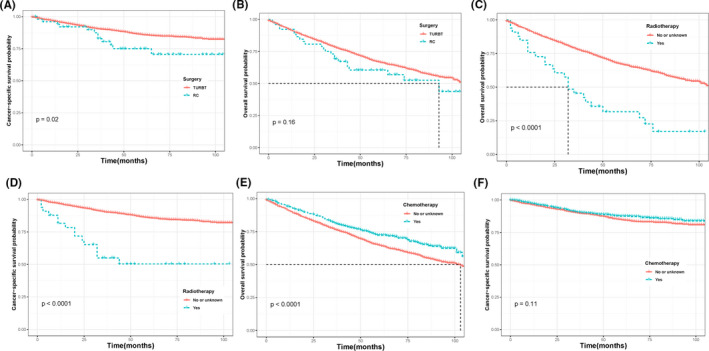
Kaplan–Meier curves of cancer‐specific survival (A, D, and F) and overall survival (B, C, and E) based on treatment of patients with BNI. BNI, bladder neck involvement

After PSM 1:1, for all T stages (Figure 2A,B), survival curves according to Kaplan–Meier showed that patients in the BNI group had bad survival comparing with those in other sites groups (*p* = 0.0056 for OS; *p* < 0.0001 for CSS). However, when stratified by T stages, subgroup analysis presented that Tis stage did not get better survival benefit for OS and CSS (*p* = 0.32, Figure 2G; *p* = 0.22, Figure 2H) and the T1 and Ta stage failed to show a significant difference in OS (*p* = 0.16, Figure 2C; *p* = 0.057, Figure 2E) while worse OSS was obtained in both T1 (*p* = 0.0027, Figure 2D) and Ta (*p* = 0.00015, Figure 2F) patients. In addition, we also stratified tumor size and tumor grade to validate the different survival outcomes between other sites and BNI cases. In patients with tumor bigger than 3 cm, BNI patients demonstrated a poorer prognosis than other sites in terms of OS (*p* < 0.001, Figure 3A) and CSS (*p* < 0.001, Figure 3B), while this difference only observed in CSS in patients with tumor size smaller than 3 cm. Similarly in the subgroup of Grade Ⅰ/Ⅱ and Grade Ⅲ/Ⅳ patients (Figure 3E–H), statistical survival differences were all showed in survive curve of OS and CSS (all *p* < 0.05). Furthermore, stratified analyses (Figure 4) by treatment like surgery, radiotherapy, and chemotherapy were also conducted to confirm the different outcomes in BNI patients. As Figure 4 showed, patients with BNI receiving radical cystectomy (*p* = 0.02 Figure 4A) and chemotherapy (*p* < 0.001 Figure 4E) had a better prognosis of OS than those not. However, we found the result that receiving radiotherapy was a passive prognosis of OS and CSS in BNI patients (all *p* < 0.001, Figure 4C,D).

## 4 DISCUSSION

This study primarily revealed that BNI was a significant predictor for OS in all T stages of NMIBC patients as well as a predictor for cancer‐special survival (CSS) (all *p* < 0.05). Moreover, patients in BNI group were at a higher risk of OM and CSM. These findings indicated that BNI was an independent risk factor for OM and CSM in patients of NMIBC.

The influence of intravesical tumor location on oncological outcome was rarely studied in patients with NMIBC. The prognosis of BNI on OS and CSS in NMIBC patients was first in detail described in our study. William T. Stephenson^9^ first proposed that the tumor's location in bladder obtained by cystoscopy was used as a predictor of survival and got the result that tumor (include NMIBC) on bladder neck had a significantly poor prognosis by survival analysis (*p* < 0.05), 5‐year survival in this population was best for ureteric orifice tumor while worst for bladder neck tumor (proportion surviving SE 0.63 ± 0.07 vs. 0.39 ± 0.07). However, the number of patients in the BNI group (*n* = 101) was not enough to get a convincing result. Moreover, when related to survival difference between subsites of bladder, it only provided survival information about 1‐, 3‐, and 5 years. The survival data obtained in this research was not adequate. Our study used the Kaplan–Meier method to reveal the difference in surviving proportion for all T stages in NMIBC patients. In addition, subgroup analysis stratified by T stage was also performed to obtain more detailed and precise information based on the results already gained. Similarly, Dutta et al^12^ investigate the prognostic significance of subsites of bladder on survival outcomes in patients with urinary bladder adenocarcinoma and got the results suggested that poorer OS were observed in tumor on bladder neck, trigone, and ureteric orifices (*p* = 0.0097). Consistent results that BNI might show poor survival and oncological outcomes were obtained in these researches. At the same time, they all failed to conduct in‐depth research because insufficient sample in their studies made their fail to detect PSM and perform subgroup analyses.

Interestingly, before PSM, a significantly higher percentage of grade III and grade IV (55.9% vs. 45.0%) was observed in the BNI group. In addition, BNI group had bigger tumor sizes (51.6% vs. 30.5%), more T1 and Tis stage patients. These discrepancies indicated that the BNI group had more inferior tumor characteristics and higher histological grades. It might be one of the fundamental reasons why the BNI group had a worse survival rate. Anatomic differences in the bladder neck were investigated in some studies.^9^ For instance, lymphatic drainage from bladder neck proceeds to the sacral nodes and median common iliac nodes, while lymphatics from other sites of bladder drain to the external and internal iliac nodes. Weiner et al.^13^ also got similar results that in patients treated with radical cystectomy tumors of bladder neck were associated with higher rates of lymph node invasion.

In addition, a retrospective report^14^ on the pathological mechanism of bladder cancer metastasis showed at the bladder neck tumors might directly invade the prostatic stroma. One of the reasons given by this research was the regular thinning of the lamina propria of the bladder neck and the anatomical differences of the intersection of the prostatic matrix and the bladder mucosa of the bladder neck.^15^ All these consequences revealed the higher invasive and metastatic potential of BNI tumor. In this paper, BNI was found to be an independent prognostic factor of OM and CSM for NMIBC patients after PSM. Since most of the current predictive model^16^, ^17^, ^18^ for NMIBC did not include tumor location, we suggested that tumors on bladder neck patients might be classified into high‐risk group, and more aggressive treatment might be considered.

In terms of survival analysis results, statistical difference both on CSS was only showed in the Ta and T1 stage between two groups. A poor oncological outcome of Tis stages was revealed in most studies.^16^, ^17^, ^18^ Risk factors might make survival differences more significant for a better disease outcome. Similarly, the better outcome in NMIBC patients explains why the dissimilarity of survival curve between the two groups was not prominent. Moreover, subgroup analysis except for subgroup of tumor size <3 cm all provided a poor prognosis of OS and CSS in patients with BNI.

Up to now, the main treatment of NMIBC patients was conservative treatment such as TURBT and intravesical chemotherapy.^19^ However, some guidelines were dedicated to picking out the high risk of NMIBC patients who needed to take more aggressive treatment which included early radical cystectomy and BCG perfusion. In this paper, BNI was confirmed as a risk factor of OS and CSS in NMIBC patients especially for the Ta and T1 stage and BNI was suggested to be a predictor of high‐risk group. Patients with BNI receiving radical cystectomy showed better OS than not receiving (Figure 4A). This result might indicate that taking early radical cystectomy made BNI patients obtain survival benefits. Moreover, patients with high‐risk tumors should undergo cystoscopy at 3 months and repeated every 3 months for a period of 2 years, and every 6 months until 5 years.^19^ Therefore, we suggested that more frequent follow‐up was needed in BNI patients.

The current study had several major strengths. First, we enrolled 19,919 patients of NMIBC from SEER database (2004–2015). This study had the largest sample in history and the most detailed clinicopathological characteristics and survival outcomes of patients with NMIBC. Thus, we had sufficient sample to conduct deep and multiform analysis. PSM was applied to perform potential confounding factors so that we could get more balanced baseline characteristics. Second, in many past studies on NMIBC,^18^, ^20^ patients in Tis stage were often excluded from research because of their different morphology and clinical characteristics. However, we did not exclude them from our study but adopted subgroup analysis to go further study. However, our study also had limitations. First, our study was of a retrospective nature with unavoidable selection bias even using PSM. Second, the SEER database lacked some important variables such as lymphatic vessel invasion, intravesical therapies, and concomitant CIS. They were all powerful and significant risk factors for disease outcomes of NMIBC. Third, 21 patients were excluded from the BNI group after 1:1 PSM. Excluding these patients to obtain more balanced baseline data might make this study less precise, although the part of patients was tiny for the overall sample. Finally, after PSM, there were still statistical differences between the two groups on race, T stage, and chemotherapy. Consequently, a prospective study with stricter inclusion conditions and a more significant number of cases was needed to verify these results.

## 5 CONCLUSIONS

The prognosis of BNI was poorer than that of other subsites of bladder for NMIBC patients. BNI was an independent risk factor for OM and CSM in patients of NMIBC, especially for the Ta and T1 stage. Therefore, BNI was an essential predictor factor for NMIBC patients. More frequent follow‐up and more suitable treatment methods should be adopted for patients with tumors on the bladder neck.

## ETHICAL APPROVAL STATEMENT

The data from SEER are publicly available and de‐identified.

## CONFLICT OF INTEREST

The authors declare no conflict of interest.

## Data Availability

The data in this article come from the SEER database. The data can be found here: https://seer.cancer.gov/data/
